# The mitochondrial genome of *Empis separata* (Diptera: Empididae)

**DOI:** 10.1080/23802359.2019.1644555

**Published:** 2019-07-23

**Authors:** Yue Liu, Naizhong Chen, Ding Yang

**Affiliations:** a College of Plant Protection, China Agricultural University, Beijing, China;; b Institute of Equipment Technology, Chinese Academy of Inspection and Quarantine, Beijing, China

**Keywords:** Mitochondrial genome, Empidinae, Phylogenetics

## Abstract

The dance fly *Empis separata* belongs to the subfamily Empidinae of Empididae. The mitogenome (GenBank accession number: MK993569) of *E. separata* was sequenced, the new representative of the mitogenome of the subfamily. The complete mitogenome is 14,961 bp totally, consisting of 13 protein-coding genes, two rRNAs, and 22 transfer RNAs. All genes have the similar locations and strands with that of other published species of Empididae. The nucleotide composition biases toward A and T, which together made up 76.3% of the entirety. Bayesian inference analysis strongly supported the monophyly of Empidoidea, Empididae, and Dolichopodidae. This result also suggested that Empidinae is the sister group to the clade of Trichopezinae.

## Introduction

Empididae is one of the largest families in Diptera with over 5,000 described species from the world (Yang et al. 2007). They capture aphids, psyllids, and coccids of Hemiptera, but also other true flies such as mosquitos, blackflies, and so on. They are widely used as a biological indicator of evaluating the quality of environment and biodiversity (Yang and Yang 2004).

The specimens of *E. separata* used for this study were collected in Zhouzhi County of Shaanxi by Jinjin Ning and identified by Ding Yang. Specimens are deposited in the Entomological Museum of China Agricultural University (CAU) with the accession number: CAU201583. The total genomic DNA was extracted from the whole body (except head) of the specimen using the QIAamp DNA Blood Mini Kit (Qiagen, Germany) and stored at −20 °C until needed. The mitogenome was amplified and sequenced as described in our previous study (Wang et al. 2016). The nearly complete mitogenome of *E. separata* is 14,961 bp (GenBank accession number: MK993569). It encoded 13 PCGs, 22 tRNA genes, two rRNA genes, and the control region could not be sequenced entirely in this study and was similar with related reports before (Li et al. 2016; Wang, Ding, et al. 2016; Wang, Wang, et al. 2016; Li et al. 2017; Zhou et al. 2017; Qilemoge et al. 2018; Gao et al. 2018). All genes have the similar locations and strands with that of other published Empididae species. The nucleotide composition of the mitogenome was biased toward A and T, with 76.3% of A + T content (A = 38.5%, T = 37.8%, C = 14.1%, and G = 9.5%). The A + T content of PCGs, tRNAs, and rRNAs is 75.0%, 77.8%, and 81.5%, respectively. The total length of all 13 PCGs of *E. separata* is 11,301 bp. Five PCGs (*NAD2*, *ATP8, NAD3, NAD5*, and *NAD6*) initiated with ATT codons, and six PCGs (*COII*, *COIII*, *ATP6*, *NAD4*, *NAD4L,* and *CYTB*) initiated with ATG codons, *NAD1* initiated with TTG as a start codon, and *CO1* initiated with TCG as a start codon. Thirteen PCGs used the typical termination codons TAA in *E. separata.*

**Figure 1.**
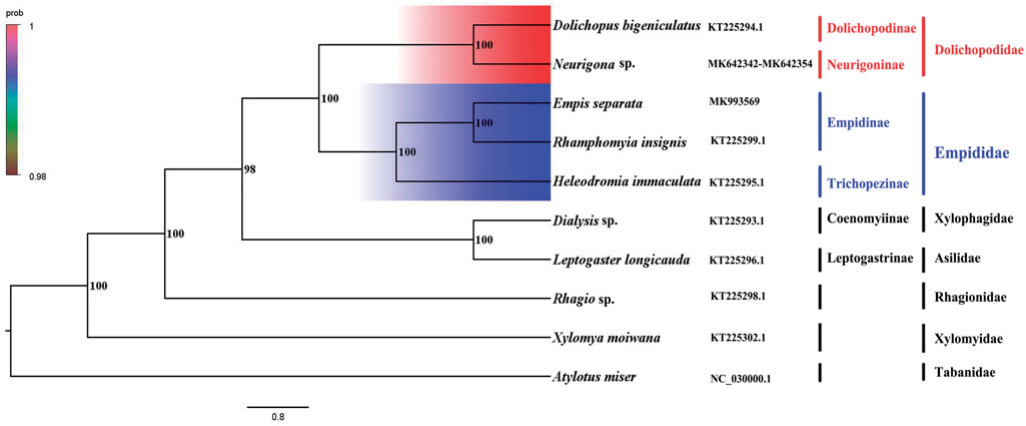
Bayesian phylogenetic tree of 10 Diptera species. The posterior probabilities are labeled at each node. Genbank accession numbers of all sequence used in the phylogenetic tree have been included in Figure 1 and corresponding to the names of all species.

Phylogenetic analysis was performed based on the nucleotide sequences of 13 PCGs from 10 Diptera species. Bayesian (BI) analysis generated the phylogenetic tree topologies based on the PCGs matrices (Figure 1). According to the phylogenetic result, the monophyly of Empidoidea was strongly supported. The monophyletic Dolichopodidae that contains Dolichopodinae and Neurigoninae was assigned to the sister group to the clade of Empididae that consists of Empidinae and Trichopezinae in this study. For the phylogeny within Empidoidea, the Empidinae is the sister to the clade of Trichopezinae, and Dolichopodinae is the sister to the clade of Neurigoninae. This result show that Dolichopodidae is the sister group to Empididae, which is consistent with the phylogenetic result of the previous research (Wang et al. 2016). The mitogenome of *E. separata* could provide the important information for the further studies of Empidoidea phylogeny.
